# Reasoning on Pore Terminology in 3D Bioprinting

**DOI:** 10.3390/gels10020153

**Published:** 2024-02-19

**Authors:** Alexander Trifonov, Ahmer Shehzad, Fariza Mukasheva, Muhammad Moazzam, Dana Akilbekova

**Affiliations:** Department of Chemical and Materials Engineering, School of Engineering, Nazarbayev University, Astana 010000, Kazakhstan; alexande.trifonov@mail.huji.ac.il (A.T.); ahmer.shehzad@nu.edu.kz (A.S.); fariza.mukasheva@alumni.nu.edu.kz (F.M.); muhammad.moazzam@nu.edu.kz (M.M.)

**Keywords:** 3D printing, hydrogel scaffold, tissue engineering, porosity, hydrogel pore nomenclature

## Abstract

Terminology is pivotal for facilitating clear communication and minimizing ambiguity, especially in specialized fields such as chemistry. In materials science, a subset of chemistry, the term “pore” is traditionally linked to the International Union of Pure and Applied Chemistry (IUPAC) nomenclature, which categorizes pores into “micro”, “meso”, and “macro” based on size. However, applying this terminology in closely-related areas, such as 3D bioprinting, often leads to confusion owing to the lack of consensus on specific definitions and classifications tailored to each field. This review article critically examines the current use of pore terminology in the context of 3D bioprinting, highlighting the need for reassessment to avoid potential misunderstandings. We propose an alternative classification that aligns more closely with the specific requirements of bioprinting, suggesting a tentative size-based division of interconnected pores into ‘parvo’-(d < 25 µm), ‘medio’-(25 < d < 100 µm), and ‘magno’-(d > 100 µm) pores, relying on the current understanding of the pore size role in tissue formation. The introduction of field-specific terminology for pore sizes in 3D bioprinting is essential to enhance the clarity and precision of research communication. This represents a step toward a more cohesive and specialized lexicon that aligns with the unique aspects of bioprinting and tissue engineering.

## 1. Introduction

Extrusion-based 3D printing technology has become a pivotal approach in tissue engineering, particularly in the fabrication of polymer-based cell-free and cell-laden constructs [1]. These constructs, designed to mimic the native extracellular matrix (ECM), play a crucial role in supporting cell viability, proliferation, and, ultimately, tissue formation. The process began with a computer-aided design (CAD) file that outlined the desired geometry of the hydrogel constructs. This file is transformed into a series of slices that guide the 3D printer in producing shapes that strictly adhere to the predetermined design. Printing involves the consecutive deposition of a specialized ink in a mesh-like pattern, forming channels within the polymer matrix. This configuration is favorable for maintaining uninterrupted nutrient and gas transport, which are key factors in tissue development while preserving the robustness of the structure.

The choice of the material for 3D bioprinting is critical. Hydrophilic polymers such as natural gelatin, alginate, and hyaluronic acid are excellent for this purpose because of their biological compatibility and shear-thinning properties [2]. First, they combine the desired biological characteristics with appropriate shear-thinning of non-Newtonian behavior, which makes them suitable for 3D printing [3]. Second, the presence of a large number of functional groups and the broad modification variability of these polymers allows the utilization of multiple crosslinking approaches to fabricate a physically stable, highly porous, interconnected hydrogel matrix that can retain its structure and integrity for durable periods and support tissue development [4,5,6,7,8,9].

Modern 3D printers, equipped with advanced electronics and software-controlled modules, offer precise mechanical control at the micrometer scale. Such precision is crucial for ensuring the geometric accuracy and structural integrity of the scaffolds, which are contingent upon the physical properties of the polymeric ink and optimized printing parameters, including extrusion speed, nozzle diameter, and temperature.

The ability of scaffolds to emulate the natural in vivo microenvironment is essential because it dictates cellular interactions and responses to mechanical stimuli from their 3D surroundings. Therefore, the material properties of scaffolds are pivotal for influencing cellular activities and tissue development [10,11,12,13,14,15,16]. Three-dimensional-printed scaffolds are typically characterized by high porosity and interconnected pore structures. The spatial distribution and geometric configuration of pores within the scaffold are important determinants of cellular penetration, proliferation, and differentiation. This, in turn, affects the deposition of the extracellular matrix, vascularization, and later stages, such as mineralization in bone tissue, ultimately influencing the formation of functional tissue.

Different pore sizes can variably influence the tissue development processes. For instance, in bone tissue engineering applications, pore sizes of approximately 100 µm are ideal for facilitating implant integration. Larger pores, on the other hand, have been found to promote angiogenesis and bone ingrowth [17,18]. In contrast, smaller pores might impede bone-implant bonding by restricting the transport of essential nutrients and oxygen. Therefore, it is necessary to strike a balance in the pore size range, typically between 100 and 700 µm [11]. This balance aims to optimize nutrient diffusion, facilitate effective cellular interactions, and maintain the structural integrity of the material.

The characterization of scaffolds in tissue engineering is a multifaceted process that involves the evaluation of key parameters such as porosity, pore diameter, elasticity, swelling capacity, and stability. However, while the majority of characteristics are assessed and reported using agreed terminology and evaluation methods, this is not the case for porosity and pore sizes in the context of hydrogels. The existing variability in terminology associated with porosity and pore size among researchers not only creates controversy but also leads to confusion within the scientific community [19,20,21,22,23]. This review addresses the current status of the terminology used in the context of hydrogels, advocating the establishment of standardized reporting conventions that meet the needs of bioprinting, tissue engineering, and related fields. The success of extrusion-based 3D printing technology in developing scaffolds for tissue engineering hinges on the precise control of scaffold geometry, selection of appropriate materials, and in-depth understanding of their interactions with cellular processes. Establishing coherent and standardized terminologies for scaffold characterization is essential for the advancement of tissue engineering research and practical applications, promoting consistent progress and innovation.

## 2. Pores in 3D-Printed Scaffolds

### 2.1. Pore Fabrication

Three-dimensional printing is frequently employed as a fabrication technique for hydrogel scaffolds for tissue engineering purposes. The standard morphological characterization of 3D-printed scaffolds includes the evaluation of two parameters: total porosity and pore size distribution, both of which are known to have an impact on the mechanical stability of the scaffold and the biological processes occurring as new tissue is formed [13,15,22,23]. Whereas the meaning of total porosity is unambiguous, there is considerable uncertainty on the matter of the term “pore” and its size classification in the literature associated with hydrogels, and in particular, 3D-printed scaffolds [24,25,26,27,28,29,30].

**Figure 1:**
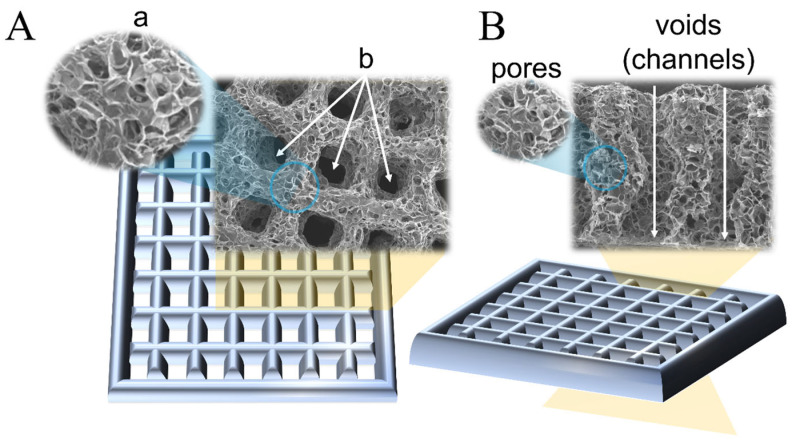
(**A**) Illustration of a rectangular scaffold with two types of morphological features: (a) interconnected porosity and (b) designed transversal openings (indicated with white arrows). (**B**) suggested terminology that allows segregation between the two types of antra: “pores” for interconnected porosity and “voids” (alternative “channels”) for the designed transversal voids. Reprinted and adapted from [8] with permission from Elsevier.

By definition, a pore is an opening that allows the passage of gases, liquids, or small molecules. Any opening that satisfies this definition is considered a pore [31]. However, this broad categorization leads to a lack of consistency in standardization across studies and applications. In scaffold fabrication, the layer-by-layer extrusion of polymeric ink in a grid-like pattern is a broadly adopted geometry for hydrogel scaffold fabrication. During extrusion, multiple transverse channels are formed (in the z-axes), enabling the efficient transport of gases and nutrients into the inner layers of the scaffold, which is critical in all tissue formation stages. These channels also contribute to maintaining the structural robustness of the scaffold [32]. Another prevalent method of scaffold fabrication is void-free printing, which employs a dual-ink system comprising a cross-linkable core ink and a sacrificial compound. Post-crosslinking, and after the sacrificial ink was washed out, the channels were revealed to be typically large and spanned hundreds of microns [32,33], ideal for ensuring the unobstructed transport of nutrients without compromising the structural integrity of the scaffold. The voids (channels) fabricated using these methods are shown in Figure 1A(b).

In addition to these engineered channels, hydrogel scaffolds are characterized by their highly interconnected porosity, resembling a sponge-like structure, as shown in Figure 1A(a). The extent of porosity and pore diameter can be controlled by manipulating various factors, including the materials used, their ratios and concentrations, the degree of crosslinking, and the synthesis conditions [12,31,34]. Both interconnected porosity and pore diameter significantly affect the kinetics and efficiency of tissue formation. This effect varies among different cell types and developmental stages [24,35,36]. 

Controlling the size of interconnected pores, particularly those beyond several tens of microns, is challenging owing to synthesis limitations and the need to maintain favorable printability characteristics. 

### 2.2. Terminology

In 3D-printed hydrogel scaffolds utilized in tissue engineering, two distinct types of openings are typically observed: (a) interconnected porosity, created during the crosslinking phase under certain conditions inside the hydrogel, and (b) strategically fabricated channels and structural pores, created as a result of patterned ink filament deposition, controlled via the 3D printer through a CAD file. Interconnected porosity characteristics can be indirectly modulated by various factors, such as the concentration and ratio of precursors, crosslinking conditions, and duration. However, these factors also influence the ink printability, robustness, and long-term stability of the scaffold; therefore, the pore size tunability is usually limited by the ink composition and its physical and chemical properties. The dimensions of interconnected pores are known to substantially influence biological processes such as cell attachment, proliferation, the kinetics and efficacy of ECM deposition, and vascularization. The typical pore diameter range used in hydrogels for tissue engineering applications varies from 1 to 250 µm. Structural pores usually have larger dimensions and are essential for ensuring the efficient diffusion of nutrients and oxygen to the center of the scaffold, thereby supporting cellular viability and tissue development equally across the scaffold. Controlling the structural pore diameter is limited by the printing accuracy characteristics and a combination of the viscoelastic properties of the ink and CAD file settings, allowing the creation of openings on a cm-scale. The two types of pores play different roles in tissue formation; however, the critical parameter is the dimensions of interconnected porosity, which has a major influence on cell behavior and evolution.

However, the literature reveals variability in the terminology employed to describe the pore sizes and their origins. For example, it is not always clear which pores the authors refer to when pore diameters are reported. Such miscommunication can be avoided by setting and using distinctive terminology when pores are mentioned. For instance, structural pores, voids, channels, axial (transversal) pores, and windows are intuitive terms for referring to a CAD-designed opening. Alternatively, pores (or interconnected pores) are more intuitive for describing the internal morphology of the hydrogels. Nonetheless, some studies employ the term “pores” ambiguously to refer to both patterned and interconnected antra, leading to confusion. Occasionally, descriptors like “bigger” and “smaller”, or the more frequently used “macro” and “micro”, are utilized to distinguish between these pore types. However, despite utilizing similar terminology, these terms are often defined differently across studies [37,38]. In this regard, adherence to distinctive terminology and the accuracy of its users facilitates communication.

Moreover, the terms “macro” and “micro”, are deeply entrenched in International Union of Pure and Applied Chemistry (IUPAC) definitions and are typically associated with pore sizes that extend beyond the scope of hydrogels. Employing the same terminology with varying definitions can be perplexing, particularly in scenarios where hydrogels serve as carriers for functional porous nanoparticles in drug delivery applications. Thus, adopting terms from the IUPAC pore size classification not only generates confusion but also constrains the use of IUPAC conventions within the same manuscript. The absence of a consensus in the terminology of hydrogel porosity poses significant challenges in understanding and comparing the experimental results. This issue is particularly pronounced for interdisciplinary scientists working at the nexus of material science, tissue engineering, and drug delivery, where the IUPAC terminology is prevalent. 

Table 1 exemplifies the use of terms for both types of pores to highlight the diversity and inconsistency of existing terminology across the literature. The definition and standardization of terms that allow simple and unambiguous understanding are essential for clear communication within and outside the community.

### 2.3. Suggested Nomenclature and Classification of Pore Sizes

To enhance the clarity in the characterization of hydrogel scaffolds, it is imperative to refine the terminology used to describe their structural features. The current practice consists of employing the terms “micro” and “macro” indiscriminately for describing pores of different dimensions and origins, the definitions of which may differ from paper to paper, sometimes by an order of magnitude. A more systematic approach would be to align the traditional meanings recognized by material scientists and the broader chemistry community. Accordingly, it would be appropriate, as it was mentioned before, for the term “pore” to be designated to describe the interconnected antra inherently present within the hydrogel matrix, whereas openings created and controlled by the CAD file should use a different term, such as “void”, “channel”, or “structural pore”. This distinction is crucial for the accurate communication and documentation of scaffold properties. Figure 1B illustrates this recommended usage, clearly differentiating “pores” from “voids” or “channels”.

The use of discipline-specific terminologies adapted to the unique characteristics and requirements of each field is a crucial aspect of scientific communication. For example, in the study of solid particles, pores are categorized based on their nature, such as inter- or intraparticle, inter-aggregation, or inter-cluster pores, each of which delineates a specific structural feature of the particles [60]. Similarly, in concrete science, a distinct classification system based on pore size and water capillary behavior has been utilized. This system segregates pores into categories such as micropores, small and medium capillaries, and entrained air, reflecting their roles in the material properties [61]. These examples highlight the diversity of pore classification systems across various disciplines.

Moreover, several size-based classifications of solid materials, as proposed by Kodiraka, Dubinin, and Cheremskoj, further illustrate the extensive range of terminologies used to describe structural features in different materials [62,63,64]. The current IUPAC recommended nomenclature, which is broadly used in all material-related disciplines, delineates pores into two primary categories: one based on the accessibility of pores to the environment, distinguishing between closed, dead-end, and open pores, and the other based on pore size, which originates from variations in N_2_ behavior during isothermal adsorption. This classification system is used for the characterization of porous solids such as catalysts, oxides, zeolites, carbon, and organic polymers, and differentiates among three main pore size ranges: micro (d < 2 nm), meso (2 < d < 50 nm), and macro (d > 50 nm) pores [32]. The division into micro-, meso-, and macropores originates in the pore-size-dependent mechanisms of molecular adsorption/desorption, which can tremendously affect the properties (e.g., catalytic) of the material. Additionally, the term “nanopores” is encountered in the literature, generally denoting pore sizes ranging from 1 to 1000 nm [65].

In the context of hydrogel materials, terms borrowed from the IUPAC nomenclature, specifically micro- and macropores, have been adopted, yet with different definitions to adapt to the micrometer pore-size scale of typical hydrogels. In some cases, pore size definitions are not commonly agreed upon, and therefore, are inconsistent. Typically, micropores in hydrogels have diameters ranging from 10 nm to 10 µm [37]. However, the definition of macropores in hydrogels varies with at least two prevailing interpretations. One definition categorizes macropores as pores > 10 µm in diameter [28,66,67,68,69], whereas others consider pores > 100 µm in diameter as macropores [54,70,71,72]. The rationale behind these divergent definitions is not explicit but is presumably rooted in the range of pore sizes typically observed in hydrogels. The use of borrowed terminology and minor inconsistencies in its application might be overlooked if micro- and nanoparticle-loaded hydrogels have not been widely researched for their potential in drug delivery systems and tissue engineering scaffolds [73,74,75,76]. However, when thorough morphological characterization is sought, maintaining uniformity in terminology is essential. Employing distinct, non-conflicting terms to describe the porosity of both the hydrogel and embedded particles could offer practical resolution. Therefore, it is advisable to adhere to either the pore size definitions recommended by the IUPAC or those tailored specifically for hydrogels. Alternatively, one could bypass the issue of terminology by directly reporting the pore dimensions.

For most hydrogel scaffolds, the pore dimensions typically range from a few to several hundred micrometers, categorizing them within the IUPAC macropore classification. However, the ambiguity surrounding the “macropore” definition, particularly when juxtaposed against the IUPAC nomenclature, has led to the proliferation of undefined and subjective descriptors in numerous reports. Terms such as “extra-large” [8,77,78], “super-large” [79,80], “ultra-large” [81,82], or “very large” [42] are often employed to underscore the exceptionally large dimensions of interconnected pores within these scaffolds. Despite their intent to convey the scale of pore size, these descriptive terms lack objective standardization, leading to significant variability in pore size interpretations among different researchers.

**Figure 2:**
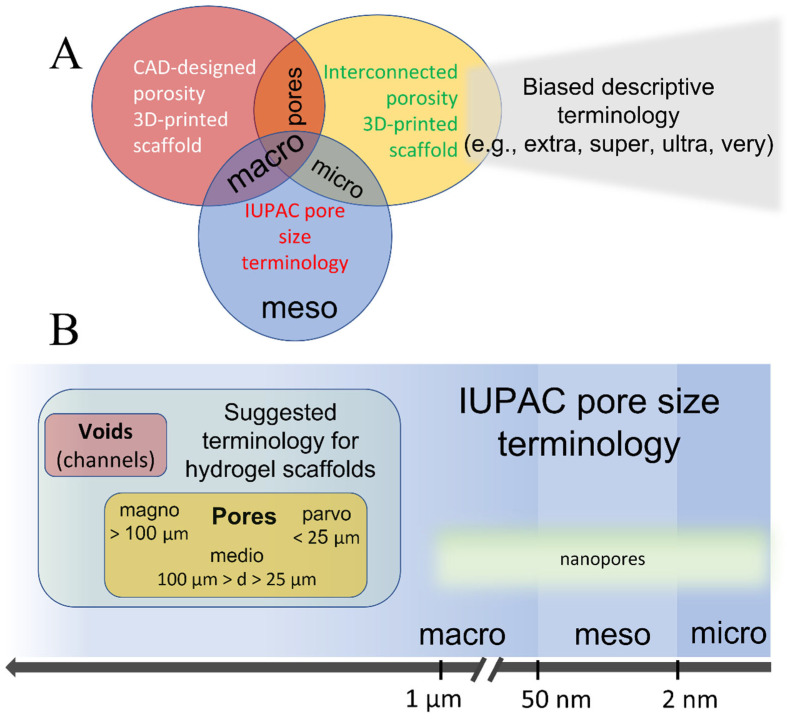
Illustrative representation of (**A**) currently used pore size terminology in 3D bioprinting and the associated overlaps with the IUPAC convention terms, and (**B**) suggested terminology for 3D-printed hydrogel as a comparison to IUPAC’s terminology.

The issue of terminology and definitions of hydrogels, as previously discussed, is undoubtedly of considerable importance and interest, particularly in the context of bioprinting [37]. The confusion stemming from the lack of a unified nomenclature coupled with the overlap with the IUPAC pore size terminology necessitates the development of a hydrogel-oriented pore size classification. Such a classification could coexist with the IUPAC-recommended nomenclature, aiding in maintaining the simplicity, clarity, and objectivity of scientific reporting. Figure 2A illustrates the points of overlapping terminologies.

To address the confusion arising from the use of “macro” and “micro” pore classes and to maintain compatibility with the IUPAC recommendations, we propose an alternative, field-specific pore classification system that categorizes pores into three distinct size ranges. This system adopts Latin-derived terminology, offering a clear and structured approach to pore-size classification. We suggest the term parvopores (derived from “parvus”, Latin for small) for pore sizes below 25 µm. For intermediate sizes, mediopores (from “medius”, meaning middle in Latin) were defined for pores ranging from 25 to 100 µm. For larger pores, the term magnopores (from “magnus”, Latin for large) is proposed, which applies to pores with dimensions greater than 100 µm. This classification not only aligns with the historical roots of scientific nomenclature but also provides a more intuitive understanding of the pore size categories.

The rationale behind this size segregation is grounded in extensive research, indicating that different pore sizes elicit varied biological responses from cells. These responses are crucial during various stages of tissue formation, influencing key processes, such as cell proliferation, ECM deposition, vascularization, and calcification [12]. Although porosity plays a crucial role in tissue formation and the interaction between cells and scaffolds, it is not the sole determinant of their effectiveness. Other factors, such as the efficiency of protein deposition, the ability of cells to migrate, and the transport of waste and gases, also significantly affect the process. Nonetheless, research has shown that specific cell types exhibit a distinct preference for certain pore sizes, which may change during different stages of tissue formation depending on the hydrogel material used. For example, it has been shown that collagen-glycosaminoglycan (CG) hydrogel scaffolds with pore sizes of 5–20 µm are optimal for neovascularization, fibroblast, and hepatocyte ingrowth, whereas pores of 20–125 µm are favorable for regeneration of mammalian skin, and 40–100 µm for osteoid ingrowth. Bone regeneration was found to be optimal in scaffolds with pores of 100–350 µm [12,83]. Yannas et al. [84] showed that skin regeneration on a CG scaffold was possible only with a mean pore size of 20–120 µm. O’Brien et al. [85] reported that cell adhesion is a surface-area-dependent process, and its efficacy decreases with increasing pore diameter. However, the advantages of 20–50 µm pores that enhance cell attachment can impose limitations at later phases, such as hindered cell proliferation and mass transport.

Our proposed classification system for pore sizes in hydrogel scaffolds was designed around the typical pore dimensions observed in these materials, acknowledging their varied roles in different stages of tissue formation, such as cell proliferation and ECM deposition. This classification spans a wide range of pore sizes, from a few micrometers to several hundred micrometers, making it applicable in various contexts. However, this definition is not absolute; to accommodate more precise size distinctions, the use of prefixes like “sub-” and “super-” is encouraged. This approach provides the flexibility needed to address specific scenarios more accurately. By adopting this classification, researchers can precisely communicate the characteristics of hydrogel scaffolds, particularly when discussing their influence on cellular behavior and tissue engineering outcomes.

To the best of our knowledge, these proposed terms have not been adopted by any existing pore size convention, making them suitable alternatives to IUPAC-borrowed terminology. Figure 2B shows the relationship between the suggested hydrogel scaffold-specific convention and IUPAC-recommended nomenclature for solid porous materials, demonstrating how these terminologies could coexist without overlap or confusion.

Moreover, the proposed terminology facilitates the use of different terms for distinct types of porosities within a single hydrogel scaffold. This classification recommends employing the terms “voids” or “channels” for the CAD-designed openings and “pores” for the naturally occurring interconnected porosity. The three pore-size divisions—parvo-, medio-, and magnopores—are not only reflective of the specific size ranges significant in tissue engineering, but also provide a clear, standardized lexicon for discussing and analyzing scaffold architectures.

The utilization of the suggested terminology allows us to eliminate confusion and ambiguity by clearly distinguishing between various pore types and sizes. This clarity is crucial for advancing research as it enables precise communication about scaffold characteristics, ensures uniformity in scientific discourse, and facilitates comparative studies. Moreover, the separation from the commonly accepted IUPAC terminology opens up the possibility for researchers to use both nomenclatures appropriately, depending on the context and requirements of their work, without any complications.

The proposal for a new size classification system for hydrogel scaffold pores, while promising, must be approached with an understanding of the inherent complexities and variability in the field. It is important to acknowledge that the relationship between pore size and biological response is not universally consistent but varies significantly depending on several factors. These include the type of cells involved, materials used in the scaffold, and specific experimental conditions under which the studies were conducted. This variability introduces a level of complexity in the establishment of a one-size-fits-all classification system.

Despite these challenges, the development of such a classification system, which proposes three (or potentially more) distinct classes of pores, offers considerable benefits. One of the primary advantages of this new system is its ability to avoid conflicts with existing commonly used nomenclature. Carefully designing a classification that is distinct from and complementary to current terminologies can provide clarity and enhance communication within the scientific community.

The proposed classification does not oversimplify the complex interactions between pore size and biological responses. Instead, it aims to provide a framework that can be used as a reference point in discussions and analyses, helping researchers categorize and compare different scaffolds more effectively.

## 3. Conclusions

In this review, we delve into the intricacies of scientific communication, focusing particularly on the ambiguities that often arise in the terminology used within the research community. These ambiguities, as it was demonstrated, can lead to confusion and complications in the interpretation and dissemination of scientific findings. The establishment of universally accepted terminology and standardized assessment approaches is paramount for fostering productive and seamless communication in the scientific world. Effective communication, characterized by clarity and precision, is instrumental in ensuring the accurate delivery of information and data analysis, which are crucial components of the scientific process.

In light of our concerns regarding the current state of pore and pore-size terminology in bioprinting, we aimed to spark a broader discussion on this subject. Adopting such a classification could facilitate a more standardized approach to reporting and analyzing data in scaffold research. This standardization is crucial for advancing the field, as it enables more precise comparisons between studies and fosters a deeper understanding of the role of scaffold architecture in tissue engineering.

Although the direct correlation between pore size and biological response may vary, the establishment of a clear and structured pore size classification system holds significant potential benefits. It promises to bring a level of standardization and clarity to the field of bioprinting and tissue engineering, aiding in the advancement of research and development of more effective scaffold-based solutions for tissue regeneration.

## Figures and Tables

**Table 1:** Pore terminology used in the characterization of hydrogel scaffolds.

Source	Specific Term Used for Designed Voids (µm)	Specific Term Used for Interconnected Pores (µm)	Method
Godoy-Gallardo et al., 2020 [39]	n/a	Small, large, and extra large pores (0–60) (60–80) (300–550)	Two-step depressurization approach
Woodfield et al., 2004 [40]	Very large pores (1640) Very small pores (150)	n/a	3D printing
Ng et al., 2018 [41]	Microstructure with pores (0.50 ± 0.13) Bigger Pores (1.67 ± 0.26)	n/a	3D printing, drop-on-demand
Han et al., 2021 [42]	Pore (50–400)	n/a	Melt Electrowritten 3D Printing
Brennan et al., 2019 [43]	Pore (100–300)	n/a	Melt Electrowritten 3D Printing
Sanchez-Salcedo et al., 2022 [44]	Ultra-large pores (450)	Macropores (20–60) Mesopores (0.0037–0.0044)	Rapid prototyping technique
Zhang et al., 2014 [45]	Macropores (400)	Mesopores (0.003–0.004)	3D Printing
Shao et al., 2020 [46]	n/a	Mesoscale pores (100–1000)	3D Bioprinting
Farzadi et al., 2015 [47]	Macropores (700)	Micropores (10–30)	3D Inkjet Printing
He et al., 2018 [48]	Macropores (445 ± 141)	Micropores (7.0 ± 1.5)	Low-Temperature Deposition Manufacturing 3D Printing
Costa et al., 2019 [49]	Macropores (400–500)	Micropores (70–100)	3D Bioplotting
Mohan et al., 2020 [50]	Macropores (>200)	Micropores (<100)	3D printing
Lin et al., 2021 [51]	Macropores (456.1 ± 11.2)	Micropores (4.44 ± 0.7)	3D printing
Liu et al., 2021 [52]	Macropores (200–800)	Micropores *	Stereolithographic 3D printing
Liu et al., 2022 [21]	Unit pores/pores/Macropores (700)	Micropores/small pores/pores (1.15 ± 0.50)	3D printing
Cox et al., 2015 [20]	Pore channels (1460–1750)	Micropores (10–60)	3D printing
Xu et al., 2016 [53]	Void region/isolated channels/pores (341.2 ± 34.2)	Micropores (40)	3D bioprinting
Egan et al., 2017 [19]	Larger pores (800)	Smaller pores (200)	3D printing
Nyberg et al., 2019 [54]	Pores/microarchitecture (200–1000)	n/a	3D printing
Ouyang et al., 2020 [34]	Interconnected void spaces/Pores/channels (450)	n/a	Void-Free 3D Printing
Seymour et al., 2021 [33]	Windows *	Interconnected voids/voids *	Microgel extrusion bioprinting
Ataie et al., 2022 [55]	Windows *	Microporosity/microscale pores (20–25)	Nanoengineered granular bioink bioprinting
Zopf et al., 2018 [56]	Micropores *	n/a	3D printing
Gupta et al., 2021 [57]	Macropores (919 ± 89)	Micropores (20–250)	Cryogenic 3D printing
Wu et al., 2019 [58]	Interconnected pores/ Macroporous (~500)	n/a	3D Bioprinting
Sultan et al., 2018 [22]	Pore/Void (500–2000)	n/a	3D printing
Kessel et al., 2020 [30]	Pore/Aperture/Mesh/Void/Macroporous (40 and 100)	n/a	3D Bioprinting
Ying et al., 2020 [26]	Macropores *	Micropores (60) Nanopores *	3D Bioprinting
Zhang et al., 2021 [59]	Macropores (~500)	n/a	3D Bioprinting

* The size is not indicated.

## Data Availability

Not applicable.
